# Stigmatizing Attitudes Towards Mental Disorders Among Non-Mental Health Professionals in Six General Hospitals in Hunan Province

**DOI:** 10.3389/fpsyt.2019.00946

**Published:** 2020-01-10

**Authors:** Qiuxia Wu, Xiaoyang Luo, Shubao Chen, Chang Qi, Winson Fu Zun Yang, Yanhui Liao, Xuyi Wang, Jinsong Tang, Yiyuan Tang, Tieqiao Liu

**Affiliations:** ^1^Department of Psychiatry, The Second Xiangya Hospital, Central South University, Changsha, China; ^2^National Clinical Research Center on Mental Disorders, Changsha, China; ^3^National Technology Institute on Mental Disorders, Changsha, China; ^4^Hunan Key Laboratory of Psychiatry and Mental Health, Changsha, China; ^5^Mental Health Institute of Central South University, Changsha, China; ^6^Department of Psychological Sciences, Texas Tech University, Lubbock, TX, United States; ^7^Female Psychiatric Ward, The First Psychiatric Hospital of Hengyang City, Hengyang, China

**Keywords:** non-mental health professionals, stigma, social distance, schizophrenia, depression, general anxiety disorder

## Abstract

**Background:** There have been few studies on the stigma associated with mental disorders among non-mental health professionals in general hospitals in China. This study seeks to explore mental health-related stigma and the desire for social distance among non-mental health professionals in general hospitals in Hunan Province in China.

**Methods:** The study was carried out with 1123 non-mental health professionals in six general hospitals in Hunan Province by using a questionnaire with a case vignette describing either schizophrenia, depression, or generalized anxiety disorder (GAD). Questions were asked about the attitudes of participants and other people towards individuals with mental disorders and the willingness to come into contact with them.

**Results:** The people described in the vignette were considered dangerous by 84.4% of participants for schizophrenia, 72.0% of participants for depression, and 63.1% of participants for GAD. Besides being dangerous, people with schizophrenia were perceived as unpredictable and as the least suitable for voting for as a politician or employing. Around 50% of participants believed the problems described in the vignette were due to personal weakness. Over 70% of the non-mental health professionals were not willing to have the people described in the vignette marry into their family. The participants had gained their mental health-related knowledge mainly through the media, mostly from newspapers.

**Conclusions:** The current study found a significant stigma towards individuals with mental disorders and a desire for social distance from such people among non-mental health professionals in general hospitals in Hunan Province. Anti-stigma interventions should focus on addressing non-mental health professionals' beliefs on dangerousness and unpredictability.

## Introduction

Stigma is a mark of shame or disgrace which sets an individual apart from others (1). It includes public stigma (attitudes from other people) and self-stigma (attitudes about self). Individuals with mental disorders usually have to struggle with the symptoms and skills deficits that arise from these disorders and the stigmatizing attitudes towards them from other people (2). Individuals with mental disorders are stigmatized when they are labeled as different from others, linked to undesirable attributes (i.e., stereotypes, such as dangerousness, unpredictability, and personal weakness), separated from others, and experience status loss and discrimination, which they usually do not have social, economic or political power to counter (3, 4). Mental health-related stigma could inhibit treatment-seeking, increase psychological distress, and adversely affect social activities and the ability to succeed in education and the workforce (5–7).

Mental health-related stigma is not only an interpersonal issue but also a health care system issue that could result in a health care crisis. There is a growing body of evidence on mental health-related stigma in the health care system and among health professionals (8). Previous studies have reported that the frequency of discrimination being experienced by individuals with mental disorders ranges from 17% to 31% in a physical health-care setting (5, 9–11). Previous research shows the average reduction in life expectancy for individuals with mental disorders ranges from 10 to 39 years compared to the general population (12–15). The mortality gap is not only driven by increased suicides and injuries but also by poor physical health, which results from the side-effects of medications and poor lifestyle. According to Daniel Vigo, years lived with disability and disability-adjusted life-years due to mental disorders were 32.4% and 13.0%, respectively (16). Furthermore, individuals with mental disorders have a higher risk of physical disorders compared to the general population (17). Therefore, individuals with mental disorders may seek treatment more frequently than the general population. When individuals with mental disorders seek physical-health treatment in a physical health-care setting, they may experience unequal, ineffective, or disrespectful treatment because of the stigmatizing attitudes towards them, which could act as a barrier to treatment-seeking by them and their family (18). Furthermore, some health professionals may attribute their physical complaints to pre-existing mental disorders, ignore the real physical conditions, and provide poor-quality health care. Some research has reported that individuals with a history of mental disorders experience poorer health care quality for their physical health conditions (19–21). The unequal treatment they experience could, in turn, increase morbidity and premature mortality (15, 22).

A great number of countries have studied mental health-related stigma based on vignettes describing a person with a certain mental disorder, including investigating the participants' attitudes about people in the vignette (known as personal stigma), their beliefs about the attitudes of others (perceived stigma), and their willingness to have social interactions with them, both among the public and health professionals (23–26). Most previous studies have focused on stigmatizing attitudes towards depression and schizophrenia, but little is known about generalized anxiety disorder (GAD). GAD is the most common anxiety in primary health care and is associated with significant disease burden (27). According to the Stigma in Global Context – Mental Health Study conducted in China, less than one-third of people could recognize schizophrenia or depression in vignettes as a mental illness, while less than 20% of people could accurately recognize the specific disease (28). Less than 5% of patients first visited psychiatrists and over 70% of patients first visited non-mental health professionals in general hospitals, according to a study conducted in a general hospital (29). Moreover, patients with depression or anxiety are more likely to present with somatic complaints rather than emotional distress (30–32). Most mental health-related facilities are psychiatric hospitals that are located in urban areas, and over 98% of mental health professionals work in psychiatric hospitals (33). Due to these factors, non-mental health professionals in general hospitals have a high probability of contact with individuals with mental disorders and play an important role in referring patients to psychiatrists in time. In China, there are few studies on mental health-related stigma conducted with non-mental health professionals (health professionals who provide health care services rather than mental health services). Given the central role health professionals play in stigma reduction campaigns and programs, it is necessary to further understand the stigma towards individuals with these common mental disorders among non-mental health professionals. The aim of the current study was to explore the stigma and social distance related to schizophrenia, depression, and generalized anxiety disorder in non-mental health professionals.

## Methods

### Sample

Data for this analysis were drawn from the survey of mental health literacy and stigma conducted in six general hospitals in Hunan province from 2014 to 2015 (34). These hospitals were Xiangya Hospital of Central South University, the Second Xiangya Hospital of Central South University, the First Affiliated Hospital of the University of South China, the First People's Hospital of Hengyang, the Fifth People's Hospital of Hengyang, and the People's Hospital of Hengyang County. These hospitals comprised four tertiary hospitals and two secondary hospitals, all of which are teaching hospitals. The convenience sampling method was applied in this study. Our previous work found that a limited number of studies concerning mental health stigma had been conducted in Hunan province. Hence, the sample size for this study was calculated based on the proportion of mental health knowledge at the chance level (50%), set at the 95% confidence interval and with 5% marginal error. Additionally, a non-response rate of 10% was included in the calculation. Based on these parameters, a sample size of 424 for each questionnaire was required. Therefore, we recruited 75 respondents for each questionnaire (three questionnaires in total) within each hospital. A total of 1350 questionnaires were distributed for the non-mental health professionals to complete on their own.

The protocol received ethical approval from the ethics committee of the Second Xiangya Hospital of Central South University. The aim of the study was clearly stated in the questionnaires, and oral informed consent was obtained from the participants.

### Survey Questionnaire

The questionnaires were based on a vignette (see **Supplementary Material**) describing a person with one of three mental disorders: schizophrenia, depression, or GAD, and adapted from the questionnaire used by Jorm et al. (35). Participants were allocated to receive one of the three vignettes on a random basis. The symptoms described in the vignettes satisfied the diagnostic criteria according to the Diagnostic and Statistical Manual of Mental Disorders, Fourth Edition (DSM-IV) and the International Classification of Diseases, Tenth Revision (ICD)-10.

After presenting the vignette, participants were required to answer a range of questions, including the most likely diagnosis, likely helpfulness of diverse interventions, likely outcomes for the person with or without treatment, likely causes and risk factors, and stigmatizing attitudes towards the person in the vignette, desire for social distance, and ways that they had learned about mental health. Data associated with these other questions are reported in our previous article (34). The main emphasis of this paper is the participants' stigmatizing attitudes towards individuals with mental disorders and desire for social distance.

#### Personal and Perceived Stigma

Stigma was estimated with two subscales (9 items each) by asking participants 1) their own attitudes (personal stigma) and 2) the participants' beliefs about most people's attitudes towards people with the problem described in the vignette (perceived stigma) (36). The perceived stigma subscale includes the same statements as in the personal stigma subscale but starts with “Most other people believe that…” Statements are beliefs such as dangerousness, unpredictability, or a sign of personal weakness. A five-point scale was used to measure the response to each item, which ranges from “strongly agree” to “strongly disagree.”

#### Social Distance

A five-item scale developed by Link et al. (37) was used to measure the willingness to come into contact (such as making friends, working closely) with the person in the vignette. A four-point scale ranging from “definitely willing” to “definitely unwilling” was used to measure response to each item.

### Statistical Analysis

Data analysis was conducted in R 3.6.1 within Rstudio 1.2.5001 (38). The median (interquartile range) and frequency (percentage) were used for demographic data, and percentage frequencies and 95% (CI) were computed for stigma and the desire for social distance. Chi-square tests and Kruskal-Wallis tests were performed to investigate whether there were any demographic differences between vignettes. For easy presentation, the categories “agree” and “strongly agree” were combined for stigma items to form a composite agreement in the analyses. For social distance items, “definitely unwilling” and “probably unwilling” categories were combined in the analysis. The detailed responses are described in **Supplementary Tables 1**–**3**. For each item on the stigma scales and social distance scale, we used the Pearson's Chi-square test to investigate whether there was any significant difference between different vignettes in the proportion of agreement.

## Results

Out of the 1350 distributed questionnaires, a total of 1123 qualified questionnaires were included in the final sample (response rate of 83.26%). The number of responses for each of the three vignettes were: schizophrenia (377), depression (372), and GAD (374). The detailed demographic characteristics of the participants are shown in **Table 1**. Over 80% of participants had at least a bachelor's degree, about 60% of them were physicians, and 54% of them were male. Over 70% of the respondents were from tertiary hospitals. The median age and work duration were 28 years old and 4 years, respectively. There were no significant differences between the demographic characteristics of respondents for the three vignettes. The rates of recognition of schizophrenia, depression, and GAD were 48.8%, 58.1%, and 31.8%, as reported in our previous paper (34).

**Table 1:** Demographic characteristics of participants responded to each vignette.

Participant characteristics	Total N = 1123		Schizophrenia N = 377		Depression N = 372		GAD N = 374		P value*
	n	%	n	%	n	%	n	%	
Gender									0.999
Male	611	54.4	204	54.1	203	54.6	204	54.5	
Female	512	45.6	173	45.9	169	45.4	170	45.5	
Age (years)^#^	28	(25-35)	28	(25-35)	27	(25-35)	28	(25-34.3)	0.882
Marriage									0.999
Married	548	48.8	185	49.1	179	48.1	184	49.2	
Unmarried	566	50.4	189	50.1	190	51.1	187	50.0	
Others (divorced, widowed)	9	0.8	3	0.8	3	0.8	3	0.8	
Specialty									0.921
Physician	676	60.2	230	61.0	223	59.9	223	59.6	
Surgeon	447	39.8	147	39.0	149	40.1	151	40.4	
Educational level									0.824
< Bachelor's degree	203	18.1	67	17.8	70	18.8	66	17.6	
Bachelor's degree	506	45.1	174	46.2	168	45.2	164	43.9	
Master's degree	347	30.9	119	31.5	109	29.3	119	21.8	
Doctor's degree	67	5.9	17	4.5	25	6.7	25	6.7	
Work duration (years)^#^	4	(2-10)	4	(2-10)	4	(2-10)	4	(2-10)	0.886
Hospital level									0.876
Tertiary hospital	807	71.9	275	72.9	273	73.4	259	69.3	
Secondary hospital	303	27.0	102	27.1	99	26.6	102	27.3	

*P-value was calculated for different vignettes, with Chi-square test for categorical variables and Kruskal-Wallis test for continuous variables.

^#^Data descriptive with median (interquartile range).

### Personal Stigma

The participants' own attitudes towards the three mental disorders are described in **Table 2**. Participants were most likely to agree with the item “people with this problem are dangerous” in all the vignettes. This was particularly notable for the schizophrenia vignette, where 84.4% participants agreed or strongly agreed with the statement. Over 60% of participants for both the depression and GAD vignettes agreed or strongly agreed that people with this problem are dangerous. Participants were least likely to agree with the statement “the problem is not a real medical illness” in the schizophrenia vignette (23.9%) and “avoid people with this problem” in the depression (13.7%) and GAD (13.9%) vignettes. Endorsement of the personal stigma items that the problem is a sign of personal weakness (51.2%) and that the person with the disorder is dangerous (84.4%), unpredictable (51.7%), or non-suitable for hiring (44.8%) or being voted for as a politician (56.8%) were highest in schizophrenia. Beliefs that the person could get rid of the problem (52.1%) and that the problem is not a real medical illness (23.3%) were highest in GAD. More than 80% of the participants showed their willingness to disclose the problem.

**Table 2:** Percentage (and 95% CI) of participants who “agree” or “strongly agree” with statements about their own attitudes towards the person in the vignette.

Statement about personal belief	Schizophrenia N = 377		Depression N = 372		GAD N = 374		P-value
	n	%	n	%	n	%	
**1. The person could snap out of the problem**	156	41.4^c^ (36.4-46.5)	175	47.0 (41.9-52.3)	195	52.1^a^ (46.9-57.3)	0.013
**2. Problem is a sign of personal weakness**	193	51.2 (46.0-56.3)	180	48.4 (43.2-53.6)	173	46.3 (41.1-51.5)	0.439
**3. Problem is not a real medical illness**	56	14.9^c^ (11.4-18.9)	70	18.8 (15.0-23.2)	87	23.3^a^ (19.1-27.9)	0.013
**4. People with this problem are dangerous**	318	84.4^bc^ (80.3-87.9)	268	72.0^ac^ (67.2-76.5)	236	63.1^ab^ (58.0-68.0)	<0.001
**5. Avoid people with this problem**	68	18.0 (14.3-22.3)	51	13.7 (10.4-17.6)	52	13.9 (10.6-17.8)	0.176
**6. People with this problem are unpredictable**	195	51.7^bc^ (46.6-56.9)	117	31.5^a^ (26.8-36.4)	107	28.6^a^ (24.1-33.5)	<0.001
**7. If I had this problem, I wouldn't tell anyone**	74	19.6 (15.7-24.0)	64	17.2 (13.5-21.4)	64	17.1 (13.4-21.3)	0.595
**8. I would not employ someone with this problem**	169	44.8^bc^ (39.7-50.0)	119	32.0^ac^ (27.3-37.0)	112	29.9^ab^ (25.3-34.9)	<0.001
**9. I would not vote for a politician with this problem**	214	56.8^bc^ (51.6-61.8)	174	46.8^ac^ (41.6-52.0)	146	39.0^ab^ (34.1-44.2)	<0.001

Symbols flagging table entries denote significant differences relative to ^a^schizophrenia; ^b^depression; ^c^GAD.

### Perceived Stigma

Participants' agreements about the items reflecting the beliefs of most other people are given in **Table 3**. Although there are slight differences in the endorsement pattern in different vignettes, participants were most likely to agree that most other people believe that the person is dangerous (>50%) and consider the problem as a sign of personal weakness (>50%). Participants tended to agree that most other people would not hire someone with this problem (>40%) and not elect a politician with this problem (>45%). These items were generally rated the highest in the schizophrenia vignette and lowest in the GAD vignette. In the schizophrenia vignette, around 60% of participants were most likely to agree that other people believe the people described in the vignette are unpredictable. Meanwhile, in the depression vignette and GAD vignette, around 50% of participants endorsed the item that the person could get rid of the problem. Between 30% and 36% of participants believed other people would not disclose the problem if they had this problem.

**Table 3:** Percentage (and 95% CI) of participants who “agree” or “strongly agree” with statements about other people's attitudes towards the person in the vignette.

Statement about others' belief	SchizophreniaN = 377		DepressionN = 372		GADN = 374		P-value
	n	%	n	%	n	%	
**1. The person could snap out of the problem**	162	43.0 ^c^ (37.9-48.1)	185	49.7 (44.5-54.9)	194	51.9^a^ (46.7-57.0)	0.039
**2. Problem is a sign of personal weakness**	198	52.5 (47.3-57.7)	191	51.3 (46.1-56.5)	188	50.3 (45.1-55.4)	0.862
**3. Problem is not a real medical illness**	90	23.9 ^b^ (19.7-28.5)	121	32.5 ^a^ (27.8-37.5)	112	29.9 (25.3-34.9)	0.027
**4. People with this problem are dangerous**	290	76.9 ^bc^ (72.3-81.1)	225	60.5^ac^ (55.3-65.5)	189	50.5^ab^ (45.3-55.7)	<0.001
**5. Avoid people with this problem**	160	42.4 ^bc^ (37.4-47.6)	119	32.0^a^ (27.3-37.0)	109	29.1 ^a^ (24.6-34.0)	<0.001
**6. People with this problem are unpredictable**	225	59.7 ^bc^ (54.5-64.7)	162	43.5^a^ (38.4-48.8)	160	42.8^a^ (37.7-48.0)	<0.001
**7. If I had this problem, I wouldn't tell anyone**	138	36.6 (31.7-41.7)	121	32.5 (27.8-37.5)	113	30.2 (25.6-35.1)	0.169
**8. I would not employ someone with this problem**	200	53.1^c^ (47.9-58.2)	179	48.1 (42.9-53.3)	163	43.6^a^ (38.5-48.8)	0.041
**9. I would not vote for a politician with this problem**	218	57.8 ^c^ (52.7-62.7)	193	51.9 (46.7-57.1)	174	46.5^a^ (41.4-51.7)	0.01

Symbols flagging table entries denote significant differences relative to ^a^schizophrenia; ^b^depression; ^c^GAD.

### Social Distance

Participants' endorsements for “probably unwilling” or “definitely unwilling” to have contact with the person described in the vignettes are shown in **Table 4**. For all of the vignettes, participants were most unwilling to marry into the family of someone with the problem (> 70%) or work closely with them (> 45%), while their willingness to spend the evening socializing tended to be higher than for other social interactions (30-40%). 30% to 50% of participants were unwilling to live next door to or make friends with the people described in the vignettes.

**Table 4:** Percentage (and 95% CI) of participants who “probably unwilling” or “definitely unwilling” to have contact with the person described in the vignette.

Social interactions	SchizophreniaN = 377		DepressionN = 372		GADN = 374		P-value
	n	%	n	%	n	%	
**Live next door**	191	50.7^bc^ (45.5-55.8)	136	36.6^a^ (31.7-41.7)	128	34.2^a^ (29.4-39.3)	<0.001
**Spend the evening socializing**	151	40.1^bc^ (35.1-45.2)	112	30.1^a^ (25.5-35.0)	113	30.2^a^ (25.6-35.1)	0.005
**Make friends**	178	47.2^bc^ (42.1-52.4)	130	34.9^a^ (30.1-40.0)	125	33.4^a^ (28.7-38.5)	<0.001
**Work closely**	207	54.9^bc^ (49.7-60.0)	172	46.2^a^ (41.1-51.5)	176	47.1^a^ (41.9-52.3)	0.04
**Marry into family**	307	81.4^bc^ (77.1-85.2)	263	70.7^a^ (65.8-75.3)	269	71.9^a^ (67.1-76.4)	0.002

Symbols flagging table entries denote significant differences relative to ^a^schizophrenia; ^b^depression; ^c^GAD.

### Participants’ Usual Sources of Mental Health Knowledge

Non-mental health professionals mostly learn their knowledge about mental health from newspapers (77%), followed by books (65%; see **Table 5**). Websites (50%) and television (38%) are also important ways to learn about mental health, although they are not utilized as much as newspapers and books. Less than 25% of the participants learn from other people's explanations.

**Table 5:** Usual source of mental health knowledge of participants.

How do you usually learn about mental health issues?	SchizophreniaN = 377		DepressionN = 372		GADN = 374	
	n	%	n	%	n	%
**Newspapers**	295	78.2 (73.7-82.3)	284	76.3 (71.7-80.6)	291	77.8 (73.3-81.9)
**Televisions**	144	38.2 (33.3-43.3)	148	39.8 (34.8-45.0)	139	37.2 (32.3-42.3)
**Websites**	185	49.1 (43.9-54.2)	188	50.5 (45.3-55.7)	194	51.9 (46.7-57.0)
**Books**	248	65.8 (60.8-70.6)	252	67.7 (62.7-72.5)	242	64.7 (59.6-69.5)
**Other people's explanations**	89	23.6 (19.4-28.2)	78	21.0 (16.9-25.5)	84	22.5 (18.3-27.0)

## Discussion

This study explored the stigma towards individuals with mental disorders among non-mental health professionals in six general hospitals in Hunan Province. The survey showed that beliefs about dangerousness, unpredictability, and signs of personal weakness, unwillingness to hire someone with this problem, unwillingness to elect a politician with this problem, and desire for social distance were universally highest for schizophrenia, while beliefs that the “person could get rid of the problem” or “the problem is not a real medical illness” were higher in GAD than in the other disorders. The findings are consistent with previous reports that individuals with schizophrenia faced higher level stigma compared to other mental disorders (23–25).

The results indicated that the patterns of personal stigma and perceived stigma varied between schizophrenia, depression, and GAD. The beliefs that people with the problem are dangerous and unpredictable were significantly higher for the schizophrenia vignette and much higher than in other studies about stigma among the pubic (23). With the development of internet media in recent years, media reports about violence related with schizophrenia may have contributed to the perception of dangerousness. Furthermore, non-mental health professionals may also have more access to news about violence that has happened in psychiatric wards than the general population. Violence against doctors in China may also contribute to the beliefs in dangerousness and unpredictability (39). According to a survey that included 316 hospitals in more than 30 provinces, violence against medical staff occurred in 96% of the hospitals. The same survey also reported that intentional hurting or even killing of medical staff happened in 63.7% of the hospitals in 2012 (39). Around 30% of these offenders have a history of mental disorders; 40% are introverted, isolated, and paranoid (39). These kinds of violence in the hospital may partly influence non-mental health professionals' beliefs regarding the dangerousness and unpredictability of individuals with mental disorders. People usually hold negative beliefs about individuals with mental disorders, especially schizophrenia, such as dangerousness and unpredictability, which result in high rates of unemployment. This study also demonstrated high endorsement of unwillingness to employ individuals with schizophrenia. A study conducted in four provinces in China reported that 66% of urban people with schizophrenia were unemployed and 89.6% of rural people with schizophrenia worked as farmers or fishermen (40). These professions are in less organized, low-skill sectors, and people holding these jobs do not have as much contact with other people in the community as with other jobs, and thus they are less discriminated against. Unemployment could further lead to social withdrawal, which increases the family burden, since most of the people with schizophrenia live with their families in China. Financial burden is the most common family burden reported by caregivers of schizophrenia patients in rural China (41).

Many non-mental health professionals in this study think that GAD is not a real medical illness or that the patients could get rid of the problem. This was higher than for other mental disorders. This is consistent with our previous result that GAD is less recognized as a mental disorder and is therefore less likely to be linked with stigmatizing attitudes (34). To our knowledge, the current survey is the first to assess stigma against GAD at a non-mental health professional level. Few studies have assessed the stigma and social distance related to GAD, especially in non-mental health professionals in general hospitals in China. According to an epidemiological study, anxiety disorders are the most common mental disorders (42). The prevalence of GAD amounts to 5.3% in urban China, and only 0.5% of these people have been diagnosed (43). The majority of individuals with anxiety or mood disorders do not seek immediate help from a mental health professional but instead visit a general medical practitioner, which would usually cause a delay in treatment of at least one month (44). People with GAD not only have functional impairments and lower quality of life but also have greater utilization of medical resources in the previous six months compared to people without GAD (43). In addition to the low recognition rate of GAD among non-mental health professionals (34), this may partly reflect the beliefs that GAD is not a real medical illness and that individuals could get rid of the problem that are held by non-mental health professionals.

In the current study, perceived stigma was universally higher than personal stigma throughout the items except for beliefs in dangerousness. Social desirability and perceived social norms may contribute to this difference (23, 45). Participants tend to give answers to meet social acceptance. Non-mental health professionals tend to believe other people would hold more stigmatizing attitudes towards individuals with mental disorders than themselves, as has been shown by the results in other studies (23, 25). It may also indicate that non-mental health professionals may be unwilling to accept their own mental disorder (if they have one), since they believe other people would discriminate against them and it may impact their career.

As with personal stigma and perceived stigma, the desire for social distance of non-mental health professionals was greatest for schizophrenia when compared with depression and GAD, which may reflect their beliefs in the dangerousness and unpredictability of people with the disorder. Participants were least willing to marry into the family across each disorder, which is in line with previous studies (23, 24). This may be related to the level of intimacy. Non-mental health professionals prefer not to have contact with the person with the disorder, such as working closely or spending an evening with them, even though less than 20% of them endorsed the item that they would avoid the person with a mental disorder in the personal stigma items. The desire for social distance increased with the level of intimacy of the activity.

The high level of stigma and social distance non-mental health professionals held in the current study may be due to a lack of knowledge and training about mental health. In the current survey, newspapers were the most common source of mental health knowledge of non-mental health professionals, which is troubling. Most undergraduate medical education programs in China have few mental health-related course hours and do not provide a clinical psychiatry clerkship and preclinical curriculum (46), which is consistent with the low mental health literacy reported in our previous paper (34). Some transferred psychiatrists in China were general medical practitioners and did not receive additional mental health-related education or training. Neither the quantity of mental health-related professionals nor the quality of mental health services is sufficient. Although the number of psychiatrists has increased during the last decades in China, there remains a shortage of mental health resources. By 2016, there were 2.20 psychiatrists and 5.42 mental health nurses per 100,000 population in China, compared to 11.9 psychiatrists and 23.5 mental health nurses per 100,000 population in high-income countries, according to the World Health Organization's Mental Health Atlas (47, 48). Furthermore, these mental health resources are distributed inequitably in urban and rural areas. People with mental health disorders in rural areas usually have limited access to mental health services: there were no psychiatrists in over 60% of counties in China (49) by 2015. The mental health-related stigma could further compound the shortage of mental health resources. Furthermore, in our previous study, less than 2% of medical students chose psychiatry as their first choice of career due to negative attitudes about psychiatry (50). Mental health resources, especially human resources, are inadequate worldwide, particularly in low-income and middle-income countries (51). In addition to training more psychiatrists, non-mental health professionals in the primary health care system could be helpful in closing the treatment gap for mental disorders. Non-mental health professionals with brief mental health-related training by mental health professionals are able to detect, diagnose, give an in-time referral and even treat individuals with mental disorders (51). Integrating mental health services into the primary care system along with general hospitals and communities is the goal of the (“686 program,”) started in 2006. Given the fact that most individuals with mental disorders first visited local general hospitals to seek help from non-mental health professionals (52), the ability of these non-mental health professionals to diagnose and refer patients and their attitudes towards them are quite important. Most non-mental health professionals believed that the problem in the vignette is a sign of weakness rather than a real medical illness and believed they could get rid of the problem, which may lead to individuals with mental disorders, especially those with non-psychotic disorders, not receiving timely referral or relevant treatment. Less than 10% of patients who first contacted non-psychiatric hospitals were diagnosed with mental disorders by their first healthcare provider, and less than 13% of them were diagnosed with mental disorders before they contacted mental health professionals (52). The high health resource expenditure before they could receive in-time referral not only increases the financial burden on patients but also occupies limited medical resources. Besides mental disorders, individuals with mental disorders have a high prevalence of physical illness due to the poor lifestyle and the side effects of psychotropic medications (53). Stigmatizing attitudes towards these patients would further inhibit treatment-seeking and affect the quality of health care (8). The poor quality of health care they received would further worsen treatment compliance, decrease psychiatric stability, shorten their life span, and reduce quality of life as well as increase family burden (53).

This study has several limitations. The major limitation is the convenience sampling we used to collect data from non-mental health professionals. Convenience sampling could induce a higher sampling error, less representativeness, and lack randomization, which may impact the validity and generalizability of our findings. Due to the convenience sampling method we used and a lack of related information, we could not adjust the clustering effects in these hospitals, which may lower the accuracy of the results. However, over 80% of the respondents had a bachelor's degree or a higher educational level, while only 57.1% of the health professionals in China have a bachelor's degree, according to the 2014 statistics (54). Moreover, more than 70% of respondents were from tertiary hospitals. We speculate that the stigma level may be higher than indicated by the results in this study, since higher-level education is associated with less stigma (55, 56). Another limitation is that around 17% of participants did not return the questionnaires or complete all the questions, which may reduce the generalizability of the results. These non-respondents may have different opinions to those of the respondents. There may also be some differences in demographic variables between non-respondents and respondents. In addition, we did not ask about non-mental health professionals' interpersonal contact with individuals with mental disorders (such as friends and family members) or their training experience regarding mental disorders. Previous studies reported that greater exposure to mental disorders and higher knowledge of mental disorder predict lower personal stigma and social distance (57, 58). Most health professionals receive at least five-year undergraduate training and a three-year standardized residency program after they complete their undergraduate training. Most non-mental health professionals in China do not have mental health training experience, especially clerkship training. Hence, the number of respondents who have mental health training may be small in our study sample.

## Conclusions

The results of this survey show a high level of desire for social distance from and stigma against individuals with mental disorders in non-mental health professionals. In addition to increasing the mental health-related course hours in medical education, mental health-related knowledge training and anti-stigma interventions regarding mental disorders among non-mental health professionals are of the utmost importance. Furthermore, the emphasis of anti-stigma interventions among non-mental health professionals should be on addressing perceptions of dangerousness and unpredictability and perception of mental disorders as a result of weakness. Since the media plays an important role in promoting mental health knowledge, the quality of disseminated knowledge is important.

## Data Availability Statement

The datasets generated for this study are available on request to the corresponding author.

## Ethics Statement

The studies involving human participants were reviewed and approved by the ethics committee of the Second Xiangya Hospital of Central South University. Written informed consent for participation was not required for this study in accordance with the national legislation and the institutional requirements.

## Author Contributions

YL, JT, XW, and TL contributed to the conception and design of the study. QW, XL, SC, and CQ organized the database. QW and WY performed the statistical analysis. QW wrote the first draft of the manuscript. WY, XW, YT, and JT revised the manuscript. TL and YT advised on the statistical analysis and interpretation of findings and reviewed drafts of the manuscript.

## Funding

This study was supported by the National Key R & D Program of China (2017YFC1310400), National Natural Science Foundation of China (Grant No. 81671324 and 81371465 to TL), and Hunan Provincial Innovation Foundation for Postgraduate (Grant No. CX2017B071 to QW).

## Conflict of Interest

The authors declare that the research was conducted in the absence of any commercial or financial relationships that could be construed as a potential conflict of interest.

## References

[1] World Health Report Mental health: new understanding, new hope. (2001) 42:1799–800.

[2] Corrigan PW Mental health stigma as social attribution. Clin Psychol Pract (2000) 7(1):48–67. 10.1093/clipsy/7.1.48

[3] Link BG Phelan JC Conceptualizing stigma. Annu Rev Sociol (2001) 27(1):363–85. 10.1146/annurev.soc.27.1.363

[4] Goffman E Stigma: Notes on the management of spoiled identity. Prentice Hall: Englewood Cliffs, NJ (1963).

[5] Lasalvia A Zoppei S Van Bortel T Bonetto C Cristofalo D Wahlbeck K Global pattern of experienced and anticipated discrimination reported by people with major depressive disorder: A cross-sectional survey. Lancet (2013) 381:55–62. 10.1016/S0140-6736(12)61379-8 23083627

[6] Schnyder N Panczak R Groth N Schultze-Lutter F Association between mental health-related stigma and active help-seeking: Systematic review and meta-analysis. Br J Psychiatry (2017) 210:261–8. 10.1192/bjp.bp.116.189464 28153928

[7] Corrigan P How stigma interferes with mental health care. Am Psychol (2004) 59:614–25. 10.1037/0003-066X.59.7.614 15491256

[8] Henderson C Noblett J Parke H Clement S Caffrey A Gale-Grant O Mental health-related stigma in health care and mental health-care settings. Lancet Psychiatry (2014) 1:467–82. 10.1016/S2215-0366(14)00023-6 26361202

[9] Harangozo J Reneses B Brohan E Sebes J Csukly G López-Ibor JJ Stigma and discrimination against people with schizophrenia related to medical services. Int J Soc Psychiatry (2014) 60:359–66. 10.1177/0020764013490263 23788438

[10] Corker E Hamilton S Henderson C Weeks C Pinfold V Rose D Experiences of discrimination among people using mental health services in England 2008-2011. Br J Psychiatry (2013) 202:58–64. 10.1192/bjp.bp.112.112912 23553696

[11] Thornicroft C Wyllie A Thornicroft G Mehta N Impact of the ‘like Minds, Like Mine' anti-stigma and discrimination campaign in New Zealand on anticipated and experienced discrimination. Aust N Z J Psychiatry (2014) 48:360–70. 10.1177/0004867413512687 24253359

[12] Wahlbeck K Westman J Nordentoft M Gissler M Laursen TM Outcomes of Nordic mental health systems: Life expectancy of patients with mental disorders. Br J Psychiatry (2011) 199:453–8. 10.1192/bjp.bp.110.085100 21593516

[13] Lawrence D Hancock KJ Kisely S The gap in life expectancy from preventable physical illness in psychiatric patients in Western Australia: Retrospective analysis of population based registers. BMJ (2013) 346:1–14. 10.1136/bmj.f2539 PMC3660620 23694688

[14] Ajetunmobi O Taylor M Stockton D Wood R Early death in those previously hospitalised for mental healthcare in Scotland: A nationwide cohort study, 1986-2010. BMJ Open (2013) 3:1–9. 10.1136/bmjopen-2013-002768 PMC3731727 23901025

[15] Thornicroft G Physical health disparities and mental illness: the scandal of premature mortality. Br J Psychiatry (2011) 199:441–2. 10.1192/bjp.bp.111.092718 22130744

[16] Vigo D Thornicroft G Atun R Estimating the true global burden of mental illness. Lancet Psychiatry (2016) 3:171–8. 10.1016/S2215-0366(15)00505-2 26851330

[17] Leucht S Burkard T Henderson J Maj M Sartorius N Physical illness and schizophrenia: a review of the literature. Acta Psychiatr Scand (2007) 116:317–33. 10.1111/j.1600-0447.2007.01095.x 17919153

[18] Clement S Schauman O Graham T Maggioni F Evans-Lacko S Bezborodovs N What is the impact of mental health-related stigma on help-seeking? A systematic review of quantitative and qualitative studies. Psychol Med (2015) 45:11–27. 10.1017/S0033291714000129 24569086

[19] Jones S Howard L Thornicroft G ‘Diagnostic overshadowing': worse physical health care for people with mental illness. Acta Psychiatr Scand (2008) 118:169–71. 10.1111/j.1600-0447.2008.01211.x 18699951

[20] Shefer G Henderson C Howard LM Murray J Thornicroft G Diagnostic overshadowing and other challenges involved in the diagnostic process of patients with mental illness who present in emergency departments with physical symptoms – A qualitative study. PloS One (2014) 9:e111682. 10.1371/journal.pone.0111682 25369130 PMC4219761

[21] van Nieuwenhuizen A Henderson C Kassam A Graham T Murray J Howard LM Emergency department staff views and experiences on diagnostic overshadowing related to people with mental illness. Epidemiol Psychiatr Sci (2013) 22:255–62. 10.1017/S2045796012000571 PMC8367326 23089191

[22] Thornicroft G Premature death among people with mental illness. BMJ (2013) 346:f2969–9. 10.1136/bmj.f2969 23674141

[23] Griffiths KM Nakane Y Christensen H Yoshioka K Jorm AF Nakane H Stigma in response to mental disorders: a comparison of Australia and Japan. BMC Psychiatry (2006) 6:21. 10.1186/1471-244X-6-21 16716231 PMC1525161

[24] Reavley NJ Jorm AF Stigmatizing attitudes towards people with mental disorders: findings from an Australian National Survey of Mental Health Literacy and Stigma. Aust N Z J Psychiatry (2011) 45:1086–93. 10.3109/00048674.2011.621061 22023236

[25] Reavley NJ Mackinnon AJ Morgan AJ Jorm AF Stigmatising attitudes towards people with mental disorders: a comparison of Australian health professionals with the general community. Aust N Z J Psychiatry (2014) 48:433–41. 10.1177/0004867413500351 23943633

[26] Subramaniam M Abdin E Picco L Shahwan S Jeyagurunathan A Vaingankar JA Continuum beliefs and stigmatising beliefs about mental illness: results from an Asian community survey. BMJ Open (2017) 7:1–10. 10.1136/bmjopen-2016-014993 PMC5594210 28381420

[27] Wittchen H-U Generalized anxiety disorder: prevalence, burden, and cost to society. Depress Anxiety (2002) 16:162–71. 10.1002/da.10065 12497648

[28] Huang D Yang LH Pescosolido BA Understanding the public's profile of mental health literacy in China: a nationwide study. BMC Psychiatry (2019) 19:20. 10.1186/s12888-018-1980-8 30642305 PMC6332702

[29] Li X Zhang W Lin Y Zhang X Qu Z Wang X Pathways to psychiatric care of patients from rural regions: a general-hospital-based study. Int J Soc Psychiatry (2014) 60:280–9. 10.1177/0020764013485364 23704113

[30] Ryder AG Yang J Zhu X Yao S Yi J Heine SJ The cultural shaping of depression: somatic symptoms in China, psychological symptoms in North America? J Abnorm Psychol (2008) 117(2):300–13. 10.1037/0021-843X.117.2.300 18489206

[31] Parker G Cheah YC Roy K Do the Chinese somatize depression? A cross-cultural study. Soc Psychiatry Psychiatr Epidemiol (2001) 36(6):287–93. 10.1007/s001270170046 11583458

[32] Hsu LK Folstein MF Somatoform disorders in Caucasian and Chinese Americans. J Nerv Ment Dis (1997) Jun 185(6):382–7. 10.1097/00005053-199706000-00004 9205424

[33] Liu C Chen L Xie B Yan J Jin T Wu Z Number and characteristics of medical professionals working in Chinese mental health facilities. Shanghai Arch Psychiatry (2013) 25:277–85. 10.3969/j.issn.1002-0829.2013.05.003 PMC4054572 24991167

[34] Wu Q Luo X Chen S Qi C Long J Xiong Y Mental health literacy survey of non-mental health professionals in six general hospitals in Hunan Province of China. PloS One (2017) 12(7), e0180327. 10.1371/journal.pone.0180327 28678848 PMC5498045

[35] Jorm AF Korten AE Jacomb PA Christensen H Rodgers B Pollitt P ‘Mental health literacy': a survey of the public's ability to recognise mental disorders and their beliefs about the effectiveness of treatment. Med J Aust (1997) 166:182–6. 10.5694/j.1326-5377.1997.tb140071.x 9066546

[36] Griffiths KM Christensen H Jorm AF Evans K Groves C Effect of web-based depression literacy and cognitive-behavioural therapy interventions on stigmatising attitudes to depression: randomised controlled trial. Br J Psychiatry (2004) 185:342–9. 10.1192/bjp.185.4.342 15458995

[37] Link BG Phelan JC Bresnahan M Stueve A Pescosolido BA Public conceptions of mental illness: labels, causes, dangerousness, and social distance. Am J Public Health (1999) 89:1328–33. 10.2105/ajph.89.9.1328 PMC1508784 10474548

[38] R Core Team R: A language and environment for statistical computing. R Foundation for Statistical Computing, Vienna, Austria (2019). URL: https://www.r-project.org/.

[39] Jia X Zhou H Zhao Y Zheng L Wei Q Zheng X Investigation on hospital violence 2003–2012 in China. Chin Hosp (2014) 18:1–3. (in Chinese).

[40] Yang LH Phillips MR Li X Yu G Zhang J Shi Q Employment outcome for people with schizophrenia in rural v. urban China: population-based study. Br J Psychiatry (2013) 203:272–9. 10.1192/bjp.bp.112.118927 PMC3796368 23258768

[41] Yu Y Liu Z-W Tang B-W Zhao M Liu X-G Xiao S-Y Reported family burden of schizophrenia patients in rural China. PloS One (2017) 12:e0179425. 10.1371/journal.pone.0179425 28628657 PMC5476254

[42] Huang Y Wang Y Wang H Liu Z Yu X Yan J Prevalence of mental disorders in China: a cross-sectional epidemiological study. Lancet Psychiatry (2019) 6:211–24. 10.1016/S2215-0366(18)30511-X 30792114

[43] Yu W Singh SS Calhoun S Zhang H Zhao X Yang F Generalized anxiety disorder in urban China: prevalence, awareness, and disease burden. J Affect Disord (2018) 234:89–96. 10.1016/j.jad.2018.02.012 29524751

[44] Thompson A Hunt C Issakidis C Why wait? Reasons for delay and prompts to seek help for mental health problems in an Australian clinical sample. Soc Psychiatry Psychiatr Epidemiol (2004) 39:810–7. 10.1007/s00127-004-0816-7 15669662

[45] Norman RMG Sorrentino RM Windell D Manchanda R The role of perceived norms in the stigmatization of mental illness. Soc Psychiatry Psychiatr Epidemiol (2008) 43:851–9. 10.1007/s00127-008-0375-4 18575793

[46] Hu X Rohrbaugh R Deng Q He Q Munger KF Liu Z Expanding the mental health workforce in China: narrowing the mental health service gap. Psychiatr Serv (2017) 68:987–9. 10.1176/appi.ps.201700002 28806895

[47] World Health Organization Mental Health Atlas-2017 country profiles. Geneva: World Heal Organization (2018). https://www.who.int/mental_health/evidence/atlas/profiles-2017/en/.

[48] World Health Organization Mental Health Atlas 2017. Geneva: World Heal Organization (2017).www.who.int/mental_health.

[49] Liang D Mays VM Hwang WC Integrated mental health services in China: challenges and planning for the future. Health Policy Plan (2018) 33(1):107–22. 10.1093/heapol/czx137 PMC5886187 29040516

[50] Wang X Xiang X Hao W Liu T Attitudes toward psychiatry as a prospective career among medical students in their pre-clinical year in China- a pilot study. PloS One (2013) 8:e73395. 10.1371/journal.pone.0073395 24023869 PMC3759458

[51] Kakuma R Minas H van Ginneken N Dal Poz MR Desiraju K Morris JE Human resources for mental health care: current situation and strategies for action. Lancet (2011) 378:1654–63. 10.1016/S0140-6736(11)61093-3 22008420

[52] Zhang W Li X Lin Y Zhang X Qu Z Wang X Pathways to psychiatric care in urban north China: a general hospital based study. Int J Ment Health Syst (2013) 7:22. 10.1186/1752-4458-7-22 24020825 PMC3852166

[53] Hert M DE Correll CU Bobes J Cetkovich-Bakmas M Cohen D Asai I Physical illness in patients with severe mental disorders. I. Prevalence, impact of medications and disparities in health care. World Psychiatry (2011) 10:52–77. 10.1002/j.2051-5545.2011.tb00014.x 21379357 PMC3048500

[54] National Health and Family Planning Commission China Health and family planning statistical yearbook 2015. Beijing: Peking union medical college press (2015).

[55] Griffiths KM Christensen H Jorm AF Predictors of depression stigma. BMC Psychiatry (2008) 8:25. 10.1186/1471-244X-8-25 18423003 PMC2386456

[56] Cook TM Wang J Descriptive epidemiology of stigma against depression in a general population sample in Alberta. BMC Psychiatry (2010) 10:29. 10.1186/1471-244X-10-29 20398429 PMC2873467

[57] Busby Grant J Bruce CP Batterham PJ Predictors of personal, perceived and self-stigma towards anxiety and depression. Epidemiol Psychiatr Sci (2016) 25:247–54. 10.1017/S2045796015000220 PMC6998700 25791089

[58] Crisp A Gelder M Goddard E Meltzer H Stigmatization of people with mental illnesses: a follow-up study within the Changing Minds campaign of the Royal College of Psychiatrists. World Psychiatry (2005) 4:106–13. PMC1414750 16633526

