# Supplementary material for: Stigmatizing Attitudes Towards Mental Disorders Among Non-Mental Health Professionals in Six General Hospitals in Hunan Province
Source: Front Psychiatry. 2020 Jan 10;10:946. doi: 10.3389/fpsyt.2019.00946 (PMC6967835; doi:10.3389/fpsyt.2019.00946)
Supplement: Supplementary file 1 [file DataSheet_1.pdf]

Supplementary vignettes

The schizophrenia vignette:
San Zhang is 24 and lives at home with his parents. He has had a few temporary jobs since finishing school but is now unemployed. Over the last six months, he has stopped seeing his friends and has begun locking himself in his bedroom and refusing to eat with the family or to have a bath. His parents also hear him walking about in his bedroom at night while they are in bed. Even though they know he is alone, they have heard him shouting and arguing as if someone else is there. When they try to encourage him to do more things, he whispers that he won't leave home because he is being spied upon by the neighbors. They realize he is not taking drugs because he never sees anyone or goes anywhere.

The depression vignette:
Si Li is 26 years old. She has been feeling unusually sad and miserable for the last few weeks. Even though she is tired all the time, she has trouble sleeping nearly every night. Si Li doesn't feel like eating and has lost weight. She can't keep her mind on her work and puts off making any decisions. Even day-to-day tasks seem too much for her. This has come to the attention of Li's boss, who is concerned about her lowered productivity.

The GAD vignette:
Wu Wang is 45 years old, and she is often worried. She worries a great deal about her job performance, her children's well-being, and her relationships with men. In addition, she worries about a variety of minor matters such as getting to appointments on time, keeping her house clean, and maintaining regular contact with family and friends. It takes Wu Wang longer than necessary to accomplish tasks because she worries about making decisions. Wu Wang has trouble sleeping at night and finds that she is exhausted during the day and irritable with her.

Supplementary tables

Supplementary Table 1. Item endorsement of statements about personal attitudes to mental disorders by vignette

Statement about personal belief	Schizophrenia (N=377)					Depression (N=372)					GAD (N=374)				
	Strongly agree	Agree	Neither agree nor disagree	Disagree	Strongly disagree	Strongly agree	Agree	Neither agree nor disagree	Disagree	Strongly disagree	Strongly agree	Agree	Neither agree nor disagree	Disagree	Strongly disagree
	n(%)	n(%)	n(%)	n(%)	n(%)	n(%)	n(%)	n(%)	n(%)	n(%)	n(%)	n(%)	n(%)	n(%)	N(%)
1. The person could snap out of the problem	38 (10.1)	118 (31.3)	119 (31.6)	85 (22.5)	17 (4.5)	41 (11.0)	134 (36.0)	136 (36.6)	54 (14.5)	7 (1.9)	44 (11.8)	151 (40.4)	122 (32.6)	46 (12.3)	11 (2.9)
2. Problem is a sign of personal weakness	25 (6.6)	167 (44.3)	107 (28.4)	69 (18.3)	9 (2.4)	20 (5.4)	160 (43.0)	112 (30.1)	72 (19.4)	8 (2.2)	21 (5.6)	152 (40.6)	126 (33.7)	68 (18.2)	7 (1.9)
3. Problem is not a real medical illness	11 (2.9)	45 (11.9)	81 (21.5)	185 (49.1)	55 (14.6)	11 (3.0)	59 (15.9)	89 (23.9)	180 (48.4)	33 (8.9)	16 (4.3)	71 (20.0)	105 (28.1)	158 (42.2)	24 (6.4)
4. People with this problem are dangerous	131 (34.7)	186 (49.3)	46 (12.2)	11 (2.9)	3 (0.8)	63 (16.9)	205 (55.1)	80 (21.5)	22 (5.9)	2 (0.5)	47 (12.6)	189 (50.5)	100 (26.7)	38 (10.2)	0 (0)
5. Avoid people with this problem	12 (3.2)	56 (14.9)	103 (27.3)	168 (44.6)	38 (10.1)	10 (2.7)	41 (11.0)	79 (21.2)	203 (54.6)	39 (10.5)	9 (2.4)	43 (11.5)	106 (28.3)	181 (48.4)	35 (9.4)
6. People with this problem are unpredictable	41 (10.9)	154 (40.8)	108 (28.6)	63 (16.7)	11 (2.9)	26 (7.0)	91 (24.5)	126 (33.9)	124 (33.3)	5 (1.3)	16 (4.3)	91 (24.3)	144 (38.5)	107 (28.6)	16 (4.3)
7. If I had this problem, I wouldn't tell anyone	10 (2.7)	64 (17.0)	103 (27.3)	151 (40.1)	49 (13.0)	9 (2.4)	55 (14.8)	106 (28.5)	159 (42.7)	43 (11.6)	6 (1.6)	58 (15.5)	109 (29.1)	163 (43.6)	38 (10.2)
8. I would not employ someone with this problem	46 (12.2)	122 (32.4)	147 (39.0)	46 (12.2)	16 (4.2)	21 (5.6)	98 (26.3)	158 (42.5)	83 (22.3)	12 (3.2)	28 (7.5)	84 (22.5)	165 (44.1)	86 (23.0)	11 (2.9)
9. I would not vote for a politician with this problem	66 (17.5)	147 (39.0)	119 (31.6)	32 (8.5)	13 (3.4)	37 (9.9)	137 (36.8)	131 (35.2)	59 (15.9)	8 (2.2)	32 (8.6)	114 (30.5)	160 (42.8)	59 (15.8)	9 (2.4)

Supplementary Table 2. Item endorsement of statements about perceived attitudes to mental disorders by vignette

Statement about Others' belief	Schizophrenia (N=377)					Depression (N=372)					GAD (N=374)				
	Strongly agree	Agree	Neither agree nor disagree	Disagree	Strongly disagree	Strongly agree	Agree	Neither agree nor disagree	Disagree	Strongly disagree	Strongly agree	Agree	Neither agree nor disagree	Disagree	Strongly disagree
	n(%)	n(%)	n(%)	n(%)	n(%)	n(%)	n(%)	n(%)	n(%)	n(%)	n(%)	n(%)	n(%)	n(%)	n(%)
1. The person could snap out of the problem	30 (8.0)	132 (35.0)	114 (30.2)	88 (23.3)	13 (3.4)	42 (11.3)	143 (38.4)	121 (32.5)	61 (16.4)	5 (1.3)	36 (9.6)	158 (42.2)	129 (34.5)	43 (11.5)	8 (2.1)
2. Problem is a sign of personal weakness	17 (4.5)	180 (47.7)	114 (30.2)	56 (14.9)	10 (2.7)	19 (5.1)	172 (46.2)	121 (32.5)	54 (14.5)	6 (1.6)	17 (4.5)	171 (45.7)	142 (38.0)	39 (10.4)	5 (1.3)
3. Problem is not a real medical illness	16 (4.2)	74 (19.6)	96 (25.5)	154 (40.8)	37 (9.8)	10 (2.7)	111 (29.8)	111 (29.8)	117 (31.5)	23 (6.2)	11 (2.9)	101 (27.0)	120 (32.1)	125 (33.4)	17 (4.5)
4. People with this problem are dangerous	101 (26.8)	188 (49.9)	61 (16.2)	23 (6.1)	4 (1.0)	49 (13.2)	176 (47.3)	96 (25.8)	49 (13.2)	2 (0.5)	37 (9.9)	152 (40.6)	132 (35.3)	51 (13.6)	2 (0.5)
5. Avoid people with this problem	34 (9.0)	126 (33.4)	120 (31.8)	83 (22.0)	14 (3.7)	15 (4.0)	104 (28.0)	124 (33.3)	113 (30.4)	16 (4.3)	18 (4.8)	91 (24.3)	145 (38.8)	99 (26.5)	21 (5.6)
6. People with this problem are unpredictable	42 (11.1)	183 (48.5)	104 (27.6)	43 (11.4)	5 (1.3)	23 (6.2)	139 (37.4)	126 (33.9)	78 (21.0)	6 (1.6)	21 (5.6)	139 (37.2)	137 (36.6)	64 (17.1)	13 (3.5)
7. If I had this problem, I wouldn't tell anyone	26 (6.9)	112 (29.7)	132 (35.0)	82 (21.8)	25 (6.6)	17 (4.6)	104 (28.0)	136 (36.6)	99 (26.6)	16 (4.3)	16 (4.3)	97 (25.9)	135 (36.1)	98 (26.2)	28 (7.5)
8. I would not employ someone with this problem	66 (17.5)	133 (35.3)	128 (34.0)	42 (11.1)	8 (2.1)	31 (8.3)	148 (39.8)	126 (33.9)	61 (16.4)	6 (1.6)	33 (8.8)	130 (34.8)	148 (39.6)	51 (13.6)	12 (3.2)
9. I would not vote for a politician with this problem	79 (21.0)	138 (36.6)	119 (31.6)	34 (9.0)	7 (1.9)	48 (12.9)	145 (39.0)	127 (34.1)	47 (12.6)	5 (1.3)	43 (11.5)	131 (35.0)	140 (37.4)	47 (12.6)	13 (3.5)

Supplementary Table 3. Item endorsement of statements social distance by vignette

	Schizophrenia (N=377)				Depression (N=372)				GAD (N=374)			
	Definitely willing	Willing	Unwilling	Definitely unwilling	Definitely willing	Willing	Unwilling	Definitely unwilling	Definitely willing	Willing	Unwilling	Definitely unwilling
	n(%)	n(%)	n(%)	n(%)	n(%)	n(%)	n(%)	n(%)	n(%)	n(%)	n(%)	n(%)
1. Live next door	49(13.0)	138(36.6)	159(42.2)	31(8.2)	51(13.7)	185(49.7)	124(33.3)	12(3.2)	55(14.7)	191(51.1)	110(29.4)	18(4.8)
2. Spend the evening socializing	48(12.7)	179(47.5)	127(33.7)	23(6.1)	62(16.7)	198(53.2)	101(27.2)	11(3.0)	58(15.5)	203(54.3)	96(25.7)	17(4.5)
3. Make friends	41(10.9)	159(42.2)	151(40.1)	26(6.9)	54(14.5)	188(50.5)	111(29.8)	19(5.1)	44(11.8)	205(54.8)	106(28.3)	19(5.1)
4. Work closely	24(6.4)	147(39.0)	164(43.5)	42(11.1)	30(8.1)	170(45.7)	144(38.7)	28(7.5)	30(8.0)	168(44.9)	145(38.8)	31(8.3)
5. Marry into family	8(2.1)	63(16.7)	166(44.0)	140(37.1)	16(4.3)	93(25.0)	173(46.5)	90(24.2)	11(2.9)	94(25.1)	186(49.7)	83(22.2)
